# News and Coming Events

**Published:** 1907-12-07

**Authors:** 


### NEWS AND COMING EVENTS.

The Broadstone Jubilee Hospital, Port Glasgow, built and equipped at a cost of over £25,000, by Mr. and Mrs. Birkmyre, Port Glasgow, in commemoration of their golden wedding, has just been opened. It is an ideally situated, excellently built, and perfectly fitted institution, possessing two large wards, accommodating ten patients each, and two smaller wards.

The first number of *The London Graduate*, the organ of the Graduates' Union, which it is hoped will be the means of keeping graduates and teachers of the London University accurately informed of matters of interest connected with the University, has just been published. It contains an excellent account of the London School of Tropical Medicine.

An interesting work is announced by the Caxton Publishing Company, of Surrey Street, London, W.C.—namely, an Atlas of Obstetrics to which the stereoscopic method has been applied throughout. The work is edited by Dr. Barbour Simpson, Senior Assistant to the Professor of Midwifery in the University of Edinburgh, and Mr. Edward Burnet, M.B., Ch.B.

The festival dinner of the London Homœopathic Hospital in aid of the building extension fund was held at the Ritz Hotel on November 20, Earl Cawdor, vice-president and treasurer, in the chair. The chairman in proposing prosperity to the hospital paid a high tribute to the successful efforts of the secretary, Mr. Attwood. A sum of £11,250 is still required to complete the extension fund of £30,000.

The London Homœopathic Hospital's appeal has met with such hearty support that only £10,000 is now required to complete the £30,000 it is estimated will be necessary to extend the hospital on its own freehold site. "Our anxiety," writes the committee, "at the present moment is that if the £10,000 still required to complete the fund by December 31st next is not obtained we lose the conditional offers of Sir Henry Tyler of £10,000 and Lord Dysart of £2,000, promised on condition that the whole of the £30,000 was raised by the end of this year."

The Cottage Hospital for Lydney and district has been carried on for thirty years past at Aylbarton, upon rented premises which were not exactly suited to the present-day requirements of an institution of this character. Fortunately the committee's concern has been met and set at rest, owing to the munificence of Mr. Richard Thomas, of the firm of Richard Thomas and Company, well-known tinplate and galvanised sheeting manufacturers, of Lydney and South Wales, who has handed over to the management the sum of £1,200 with which to carry out the scheme.

Mr. Gordon Taylor, M.S., F.R.C.S., has been appointed assistant surgeon to the Middlesex Hospital.

Mr. C. A. R. Nitch, M.S.Lond., F.R.C.S.Eng., has been appointed surgeon to out-patients at St. Thomas's Hospital.

Captain H.S.H. Prince Alexander of Teck, G.C.V.O., D.S.O., etc., will take the chair at the next meeting of the Special Appeal Committee of the Royal Waterloo Hospital, to be held on Wednesday, December 11, at 11.30 a.m., at Belfast Chambers, 156 Regent Street, W. This being the last meeting for the year 1907, a large attendance of members is expected. Meetings are held monthly on the fourth Wednesday at 11.30 a.m., the next following being on Wednesday, January 22, 1908.

The Annual Conversazione and Exhibition of New Apparatus, heretofore held under the auspices of the late British Electro-Therapeutic Society, but now under the Electro-Therapeutical Section of the Royal Society of Medicine, will be held in the Queen's (small) Hall on Friday, December 13, 1907, from 7.30 p.m. to 11 p.m. The Exhibition will be open from 3 p.m., and light refreshments will be provided both afternoon and evening. All the leading makers of electro-medical and $x$-ray apparatus are taking part, and many new designs will be shown—as far as possible under working conditions. A noteworthy feature will be a display of $x$-ray tubes, from the earliest to the latest patterns. Medical practitioners will be welcome on presentation of their visiting-cards, as also will members of the dental profession and recognised teachers of the physical sciences. Others will be admitted on payment of a fee of 5s. Communications regarding cards of admission or other matters must be addressed to Reginald Morton, M.D., Hon. Secretary, 22 Queen Anne Street, Cavendish Square, London, W.

